# The future of healthcare‐associated infection surveillance: Automated surveillance and using the potential of artificial intelligence

**DOI:** 10.1111/joim.20100

**Published:** 2025-06-05

**Authors:** Suzanne D. van der Werff, Stephanie M. van Rooden, Aron Henriksson, Michael Behnke, Seven J. S. Aghdassi, Maaike S. M. van Mourik, Pontus Nauclér

**Affiliations:** ^1^ Department of Medicine Solna Division of Infectious Diseases Karolinska Institutet Stockholm Sweden; ^2^ Department of Infectious Diseases Karolinska University Hospital Stockholm Sweden; ^3^ Department of Epidemiology and Surveillance Centre for Infectious Disease Epidemiology and Surveillance National Institute for Public Health and the Environment (RIVM) Bilthoven the Netherlands; ^4^ Department of Computer and Systems Sciences Stockholm University Stockholm Sweden; ^5^ Institute of Hygiene and Environmental Medicine Charité—Universitätsmedizin Berlin Corporate Member of Freie Universität Berlin and Humboldt‐Universität zu Berlin Berlin Germany; ^6^ Berlin Institute of Health BIH Biomedical Innovation Academy BIH Charité Digital Clinician Scientist Program Charité—Universitätsmedizin Berlin Berlin Germany; ^7^ Department of Medical Microbiology and Infection Prevention University Medical Centre Utrecht Utrecht the Netherlands

**Keywords:** artificial intelligence, automated surveillance, early prediction, healthcare‐associated infections, sepsis

## Abstract

Healthcare‐associated infections (HAIs) are common adverse events, and surveillance is considered a core component of effective HAI reduction programmes. Recently, efforts have focused on automating the traditional manual surveillance process by utilizing data from electronic health record (EHR) systems. Using EHR data for automated surveillance, algorithms have been developed to identify patients with (ventilator‐associated) pneumonia and (catheter‐related) bloodstream, surgical site, (catheter‐associated) urinary tract and *Clostridioides difficile* infections (sensitivity 54.2%–100%, specificity 63.5%–100%). Mostly methods based on natural language processing have been applied to extract information from unstructured clinical information. Further developments in artificial intelligence (AI), such as large language models, are expected to support and improve different aspects within the surveillance process; for example, more precise identification of patients with HAI. However, AI‐based methods have been applied less frequently in automated surveillance and more frequently for (early) prediction, particularly for sepsis. Despite heterogeneity in settings, populations, definitions and model designs, AI‐based models have shown promising results, with moderate to very good performance (accuracy 61%–99%) and predicted sepsis within 0–40 h before onset. AI‐based prediction models detecting patients at risk of developing different HAIs should be explored further. The continuous evolution of AI and automation will transform HAI surveillance and prediction, offering more objective and timely infection rates and predictions. The implementation of (AI‐supported) automated surveillance and prediction systems for HAI in daily practice remains scarce. Successful development and implementation of these systems demand meeting requirements related to technical capabilities, governance, practical and regulatory considerations and quality monitoring.

## Graphical abstract

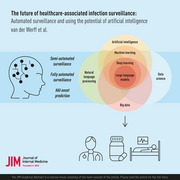

## Introduction

Artificial intelligence (AI) solutions are increasingly used for infectious disease surveillance as tools for identifying and analysing complex data structures, detecting outbreaks, classifying pathogens and assessing risk of infection, for example, in COVID‐19 and sepsis [1]. As healthcare providers move to digitalize medical data, exemplified by the use of electronic health record (EHR) systems, the stored information can be reused for automated surveillance [2] and early prediction of infections [3].

Healthcare‐associated infections (HAIs) are a common adverse event in the course of patient treatment [4, 5], associated with increased morbidity, mortality and cost [6, 7, 8]. In the European Union and European Economic Area, there are 8.9 million HAI episodes per year [4], and the burden of the six most common HAIs surpasses that of most other communicable diseases [7]. In acute care settings, the most frequently occurring HAIs are hospital‐acquired pneumonia (HAP), ventilator‐associated pneumonia (VAP), surgical site infections (SSI), urinary tract infections (UTI), catheter‐associated UTI (CAUTI), bloodstream infections (BSI), catheter‐related BSI (CRBSI) and *Clostridioides difficile* infections (CDI) [4, 5, 9–11]. A recent report by the World Health Organization estimated that as much as 70% of HAI could be prevented through adequate infection prevention measures [7, 12], illustrating the importance of infection prevention and control (IPC) programmes and early prediction for at‐risk patients. HAI surveillance is a core component of IPC programmes [13] as it has been repeatedly demonstrated that HAI surveillance can effectively reduce HAI rates [14, 15, 16].

The integral components of HAI surveillance are as follows: (1) identification of the surveillance population; (2) systematic observation; (3) ascertainment and classification of HAI based on specific case definitions that include symptoms, microbiological findings (e.g., positive blood culture) and further diagnostics (e.g., radiological findings) and (4) aggregation of outcomes into an actionable report. Hitherto, HAI surveillance has primarily been a manual exercise of reviewing patient files at most institutions. This process is associated with high demands for resources and interrater variability (differential misclassification) [17]. In recent years, there has been a focus on automating HAI surveillance to overcome these issues [2]. AI‐based methods may further improve this process, making surveillance more resource‐efficient and promoting scaling up of the implementation of surveillance activities.

This review aims to introduce important concepts for automating the critical steps of surveillance and early identification of HAI and other important infections in healthcare (where sepsis is used as an example) and to improve understanding of the potential of AI‐based methods in automated HAI surveillance. We discuss the efforts made to automate the surveillance process for several HAIs and sepsis, both with and without AI‐based methods. We also discuss the next steps in the early identification and prediction of infections. Furthermore, basic principles and focus areas for the successful implementation of reliable automated (AI‐supported) HAI surveillance are highlighted.

## Automated HAI surveillance and the use of AI

### History and concepts of automated HAI surveillance and AI

**Fig. 1:**
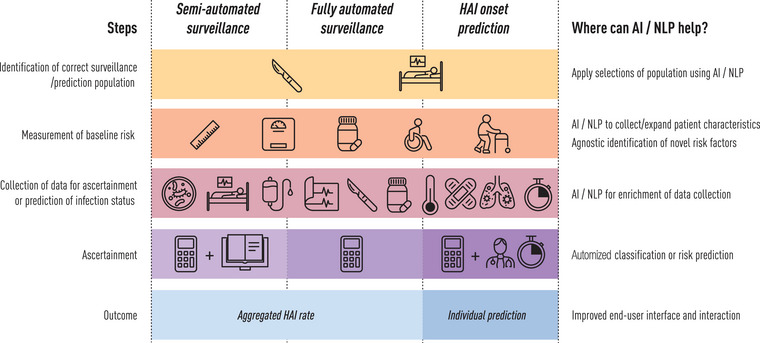
Overview of steps in the surveillance and prediction process that can be automated and where artificial intelligence (AI)/natural language processing (NLP) can assist.

For many decades, surveillance to detect existing infection events was based on manual chart review according to standardized case definitions, sometimes complemented by discussions with professionals. Subsequently, surveillance outcomes can be compared with historical rates and other healthcare facilities to identify opportunities for improvement. This time‐consuming endeavour has been increasingly replaced by automated processes and has been further facilitated by the introduction of EHR systems from the 1990s onwards [18]. There are several approaches to automating the HAI surveillance process, including selection of the surveillance population, identification of patients at risk of HAI and ascertainment of HAI status for the collection of data necessary to calculate the baseline risk (Fig. 1). In all approaches, the aim of automated surveillance is to improve standardization and reduce the workload by replacing manual decision steps with an objective and rapid automated process.

When focusing on detection of infections, a distinction is commonly made between fully and semi‐automated (or trigger‐based) surveillance [2, 19]. Fully automated surveillance implies that HAI is identified independently by a computer system, completely eliminating the need for a human review of the respective dataset. In semi‐automated surveillance, patients with a high probability of HAI are flagged using an algorithm that relies on classification rules or point‐scoring systems. Subsequently, only these pre‐selected patients are manually reviewed, thereby drastically reducing the manual ascertainment workload. In this approach, all other patients are classified as free‐of‐HAI [20].

**Fig. 2:**
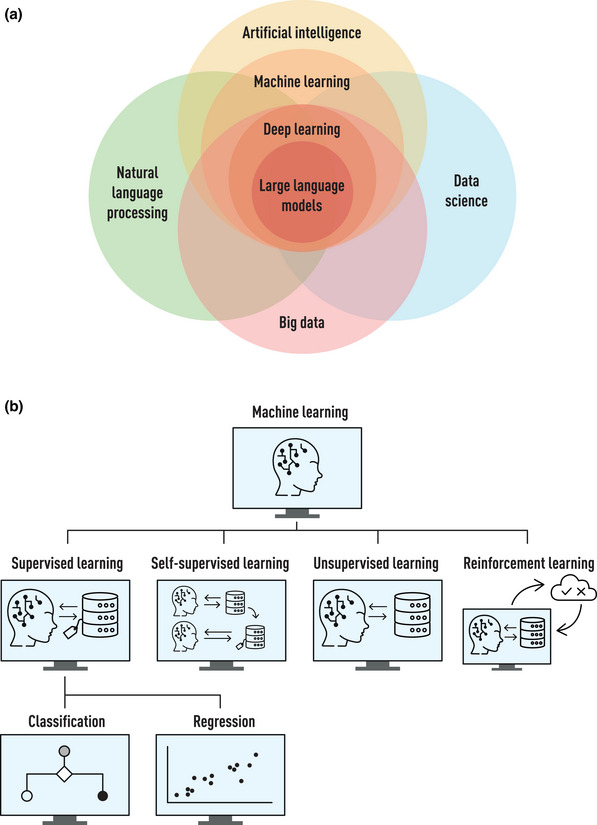
Overview of (a) relevant concepts related to artificial intelligence and how they relate to each other and (b) types of machine learning with different levels of labelled outcomes to train the models where results can be categorical (classification algorithms) or continuous (regression algorithm). For definitions kindly refer to Table 1.

Automated surveillance efforts began with local hospital‐based systems in the United States and Europe [21, 22, 23, 24, 25], often reusing data in structured repositories. Early models relied on coded data, such as diagnosis and procedure codes. In the past 10–20 years, an increasing amount of clinical data has been incorporated, including microbiology testing results, antibiotic use and vital parameters [26, 27]. Automated surveillance systems currently in use employ data science methods to support surveillance by deriving knowledge from structured clinical or administrative data. This automation approach requires the availability of the source data in a machine‐readable format. With the increasing amount and complexity of data, combined with the increased availability of computational power, the use of AI has emerged as an important technique in data science. The application of AI in this context can support multiple steps in the surveillance process. Understanding the key concepts and model development methods in the fields of AI and automated HAI surveillance is important for healthcare professionals who want to use them for IPC or clinical purposes. Fig. 2 and Table 1 visualize and summarize, respectively, concepts relevant to understanding the field of AI for the automated surveillance and prediction of HAI and other important infections.

**Table 1:** Important concepts for artificial intelligence and automated surveillance in the context of healthcare‐associated infections.

Term	Definition/explanation
Relevant concepts related to artificial intelligence (AI) (Fig. 2a)	
Data science (DS)	DS is a multidisciplinary field that uses different scientific methods to extract insights and knowledge from structured and unstructured (big) data. It combines expertise and tools from various fields such as statistics, mathematics, computer science and domain‐specific knowledge to analyse and interpret complex datasets and generate data‐driven solutions
Machine learning (ML)	ML is a subset of AI that involves using algorithms for training models to learn patterns from data and make predictions or decisions without being explicitly programmed
Deep learning (DL)	DL is a subset of ML that involves artificial neural networks with multiple layers (deep neural networks) to model complex patterns, often used in tasks like image recognition and NLP
Natural language processing (NLP)	NLP is the application of computational techniques to understand, interpret, translate and generate human language and speech (linguistics). Nowadays NLP often applies AI techniques and especially ML to achieve this goal
Large language model (LLM)	A LLM is a DL model that has been trained using very large datasets and obtains capabilities for general‐purpose language understanding and text generation
Big data	Big data refers to data that is characterized by massive **volume**, big **variety** of types and sources and high **velocity** of generation and is too complex to be processed by traditional data analysis tools. Big data often requires advanced techniques for storage, processing and analysis
Types of machine learning (Fig. 2b)	
Supervised learning	Supervised learning is a type of ML where the model is trained on (manually) labelled data, i.e., the input data comes with an output in the form of one or more labels. The model learns from these input‐output pairs to discover patterns, structures or clusters within the data. When the output or outcome is in the form of discrete labels or categories, the model is trained to perform a classification task. When the output or outcome is a continuous numerical value, the model is trained to perform a regression task
Self‐supervised learning	Self‐supervised learning is a type of ML where the model is trained using the inherent structure or characteristics of the data itself to generate the labels or supervisory signals to discover patterns, structures or clusters within the data
Unsupervised learning	Unsupervised learning is a type of ML where the model learns from unlabelled data to discover patterns, structures or clusters within the data
Reinforcement learning	Reinforcement learning is a type of ML where a model learns by interacting with an environment and receiving feedback in the form of rewards or punishments such that it leads to maximum reward to discover patterns, structures or clusters within the data

AI is a broad umbrella term that covers many aspects, and it can mean different things to different persons. Herein, AI is considered the application of advanced algorithms and models, including machine learning (ML), which enables machines to perform tasks that require some form of intelligence. In healthcare and automated surveillance, this often involves simulating human cognition when analysing complex medical data. Although there is great interest and debate surrounding the possibilities of developing artificial general intelligence, also known as strong or general AI, most AI research has been devoted to fairly narrow applications in limited, well‐defined domains, referred to as narrow or weak AI. This is also true for the application of AI in healthcare, including automated HAI surveillance.

Early forms of AI‐supported systems were typically rule‐based, that is, based on a set of if‐then rules or other heuristics defined by clinical domain experts. Although many surveillance solutions and decision support systems are still rule‐based [27, 28, 29], modern AI tends to rely on the use of ML and, in particular, deep learning (DL) for the emulation of human interpretation. This involves the use of artificial neural networks with multiple processing layers. DL generally requires larger amounts of data but can handle more input variables than less advanced models. There are different types of ML that can broadly be categorized into supervised learning, self‐supervised learning, unsupervised learning and reinforcement learning based on the degree of labelling of the outcomes in the training phase—where labelling means tagging data with the correct answers or categories to guide learning. Supervised learning remains the most commonly used type of ML in healthcare, meaning that high‐quality labelled data must be available or created. ML is the dominant approach in natural language processing (NLP), the latter of which is key to developing an AI capable of processing, understanding and generating natural language data. The use of self‐supervised learning has allowed for the pre‐training of large language models (LLMs), in the form of DL models, on massive amounts of text data [30] (Zhao et al. arXiv:2303.18223). AI‐supported systems in healthcare may incorporate NLP technologies, including (but not limited to) LLMs, to exploit the enormous amounts of EHR data recorded in free text, such as clinical notes [31, 32, 33]. If labelled data are available, a pretrained LLM can be fine‐tuned to process the free‐text better. However, if labelled data are not available, a pretrained LLM can still be used by providing instructions or examples—a method known as prompting—to help guide the LLM in performing specific tasks. Combined with more structured EHR data, this opens up the possibility of developing multimodal models that combine information recorded in different modalities to produce more accurate detection and prediction [34, 35]. Currently, certain steps in the manual surveillance process can be automated by utilizing available data from EHR systems and other electronic laboratory, hospital or patient databases, and AI can play an additional role in this automation process (Fig. 1).

First, NLP can be employed to extract structured information from unstructured clinical notes. This is particularly relevant when the information is not documented in a structured format during routine care. Especially clinical signs and symptoms such as fever, chills and inflammation of the surgical site are generally not documented in a structured manner and may benefit from the use of NLP [36, 37, 38]. Recently, several studies have attempted to use NLP to improve the correct classification of HAI; however, the success of these methods varies, and they have not yet been employed on a larger scale than individual hospitals [36, 39, 40]. Another possible application of NLP may be in the collection of information necessary for risk adjustment and interpretation of infection rates, such as classifying the indication for the surgical procedure, determining whether procedures were previously performed at the same anatomical site or assessing the pre‐operative wound class [41].

Commonly used methods of automated surveillance focus on the use of classification models or point‐scoring systems to assess the presence of HAI. This can be performed either in a semi‐automated setting, where human cognition executes the final ascertainment, or in a fully automated setting. In the latter, the case definition is generally adapted to the availability of structured source data, and information on clinical signs and symptoms is often removed; for example, in quality metrics such as Ventilator‐Associated Event (VAE) or Adult Sepsis Event (ASE) [42, 43]. A possible disadvantage of fully automated surveillance based on such a modified definition is the loss of clinical interpretability and, hence, reduced acceptance and support from healthcare professionals, which potentially limits the usefulness of the outcomes as drivers of quality improvement [20]. AI‐based methods can contribute to fully automated surveillance without requiring extensive modification of case definitions. Examples of AI‐supported surveillance of SSI, UTI and other conditions have been published in recent years [44, 45, 46, 47]. Methods such as fuzzy logic, using degrees of truth instead of the Boolean logic of true or false [48], or uncertainty quantification methods in ML models may be able to incorporate fewer black‐and‐white scenarios compared with traditional rule‐based models, thereby more closely emulating clinical practice.

### Automated HAI surveillance and use of AI for common and important HAI

Efforts have been made to automate the surveillance of several HAIs and other important infections in healthcare, both with and without utilizing AI‐based methods. Examples of studies for common HAI and other infections are summarized in Table 2. There are no clear cut‐offs for determining when an algorithm is sufficiently accurate for a specific task. However, severe infections and semi‐automated surveillance generally require algorithms with high sensitivity, whereas less severe infections and fully automated surveillance necessitate high specificity to achieve clinical acceptance and surveillance usefulness. Most studies have not used advanced AI and include rule‐based algorithms; the few studies that used advanced AI mainly applied NLP. Therefore, it is not yet possible to assess whether and when certain AI methods outperform others or which are most applicable in which setting. However, the full potential of AI in HAI surveillance has not yet been explored.

**Table 2:** Examples of previous studies on automated surveillance of healthcare‐associated infections and other important infections in healthcare.

Infection	First author [reference]	Year of publication	Type of surveillance	Phase	Year of study	Country	Hospital population	Study size^a^	Definition/reference infection	AI method used^b^	Type of algorithm^b^	Source data used by algorithm^b^	Performance metrics^b^
BSI	Bellini [49]	2007	Fully automated	Development and validation	N/A	Switzerland	General, adults	100 episodes with positive blood cultures	CDC/NHSN BSI	N/A	Rule‐based	Administrative data Microbiology results	Sens: 96% Spec: 94% Acc: 95%
BSI	Bouzbid [50]	2011	Fully automated	Development and validation	2000–2006	France	ICU, adults	1499 patients	HELICS BSI	N/A	Rule‐based	Administrative data Microbiology results	Sens: 79.6% Spec: 97.4% PPV: 50.6% NPV: 99.3%
BSI	De Bus [51]	2014	Semi‐automated	Development and validation	2011–2012	Belgium	ICU, adults	876 admissions	CDC/NHSN BSI	N/A	Rule‐based	Administrative data Microbiology results Vital parameters Medication prescriptions	Sens: 100% Spec: 100% Acc: 100% Kappa: 0.96
BSI	Redder [52]	2015	Fully automated	Validation	2010–2014	Denmark	General	925 patients	CDC/NHSN BSI	N/A	Rule‐based	Administrative data Microbiology results Medication prescriptions	Sens: 100% Spec: 100% PPV: 88% NPV: 100%
CRBSI	Trick [22]	2004	Semi‐automated	Development and validation	2001–2002	United States	General, adults	135 positive blood cultures from 127 patients	CDC/NNIS CLABSI	N/A	Rule‐based	Administrative data Microbiology results Medication prescriptions CVC data	Sens: 81% Spec: 90% PPV: 81% NPV: 90% Kappa: 0.73
CRBSI	Bellini [49]	2007	Fully automated	Development and validation	N/A	Switzerland	General, adults	351 nosocomial BSI episodes	CDC/NHSN CLABSI	N/A	Rule‐based	Administrative data Microbiology results	Sens: 78% Spec: 93% Acc: 82% Kappa: 0.72
CRBSI	Woeltje [53]	2008	Semi‐automated	Development and validation	2005–2006	United States	ICU, adults	771 blood culture isolates from 540 patients	CDC/NHSN CLABSI	N/A	Rule‐based	Administrative data Microbiology results CVC data	Sens: 94.3% Spec: 68.0% PPV: 22.8% NPV: 99.2%
CRBSI	Woeltje [54]	2011	Fully automated	Development and validation	2005–2006	United States	Non‐ICU, adults	391 positive blood cultures from 331 patients	CDC/NHSN CLABSI	N/A	Rule‐based	Administrative data Microbiology results Vital parameters CVC data	Sens: 95.2% Spec: 97.5% PPV: 90.0% NPV: 99.2% Kappa: 0.908
CRBSI	Bouzbid [50]	2011	Fully automated	Development and validation	2000–2006	France	ICU, adults	806 patients	HELICS CRI	N/A	Rule‐based	Administrative data Microbiology results	Sens: 92.0% Spec: 70.8% PPV: 17.2% NPV: 99.3%
CRBSI	Kaiser [55]	2014	Semi‐automated	Development and validation	2009–2010	Netherlands	ICU, adults	553 patients	CDC/NHSN CLABSI	N/A	Rule‐based	Administrative data Microbiology results Vital parameters Medication prescriptions CVC data	Sens: 91.7% Spec: 100% PPV: 100% NPV: 99.6%
CRBSI	Snyders [56]	2015	Fully automated	Development and validation	2011	United States	ICU, adults	643 positive blood cultures from 518 patients	CDC/NHSN CLABSI	N/A	Rule‐based	Administrative data Microbiology results Vital parameters CVC data	Sens: 81.6% Spec: 96.2% PPV: 57.4% NPV: 98.8% Kappa: 0.650
CRBSI	Karmefors Idvall [57]	2024	Fully automated	Development and validation	2017–2019	Sweden	General	181,354 episodes from 132,850 admissions	ECDC CRI3‐CVC	N/A	Rule‐based	Administrative data Microbiology results	Sens: 88.0% Spec: 100% PPV: 91.8% NPV: 100% AUROC: 0.940 Kappa: 0.899
Sepsis	Rhee [58]	2017	Fully automated	Validation	2014	United States	General, adults	510 admissions	Sepsis‐3 (overall)	N/A	Rule‐based	Administrative data Microbiology results Clinical chemistry Medication prescriptions Diagnosis codes	Sens: 69.7% Spec: 98.1% PPV: 70.4% NPV: 98.0%
Sepsis	Valik [59]	2020	Fully automated	Development and validation	2012–2013	Sweden	Non‐ICU, adults	82,653 admissions	Sepsis‐3 (overall and hospital‐onset)	N/A	Rule‐based	Administrative data Microbiology results Vital parameters Clinical chemistry Medication prescriptions Diagnosis codes	*Overall* Sens: 88.7% Spec: 98.5% PPV: 88.1% NPV: 98.6% *Hospital‐onset* Sens: 79.4% Spec: 99.7% PPV: 87.7% NPV: 99.4%
SSI	Perdiz [60]	2016	Semi‐automated	Development and validation	2009–2012	Brazil	Surgical (THA and TKA), adults	281 surgeries	CDC/NHSN SSI	N/A	Rule‐based	Administrative data Medication prescriptions	*THA* Sens: 100% Spec: 87.1% PPV: 26.7% NPV: 100% *TKA* Sens: 100% Spec: 94.6% PPV: 64.3% NPV: 100%
SSI	Sips [23]	2017	Semi‐automated	Development and validation	2004–2010 and 2012–2014	Netherlands	Surgical (THA and TKA), adults	1637 surgeries	National SSI (similar to ECDC SSI)	N/A	Point‐scoring system	Administrative data Microbiology results Surgical procedures Medication prescriptions	Sens: 100% PPV: 68.2% WR: 97.3%
SSI	Verberk [61]	2020	Semi‐automated	External validation	2012–2018	Netherlands	Surgical (THA and TKA), adults	8378 surgeries	CDC/NHSN SSI	N/A	Point‐scoring system	Administrative data Microbiology results Surgical procedures Medication prescriptions	Sens: 93.6%–100% PPV: 55.8%–72.2% WR: 98.0%–98.5%
SSI	Van Rooden [62]	2020	Semi‐automated	Development and validation	2017–2019	Netherlands, France and Spain	Surgical (THA and TKA, cardiac and colon), adults	13,723 surgeries	CDC/NHSN SSI (or similar)	N/A	Point‐scoring system	Administrative data Microbiology results Surgical procedures Medication prescriptions Radiology procedures	*THA and TKA* Sens: 81.8%–100% PPV: 17.4%–62.5% WR: 92.2%–97.5% *Cardiac* Sens: 66.7%–100% PPV: 7.9%–34.8% WR: 73.2%–96.2% *Colon* Sens: 83.7%–100% PPV: 16.6%–36.1% WR: 72.3%–82.2%
SSI	Wu [63]	2023	Fully automated	Development and validation	2013–2020	Canada	Surgical (THA and TKA), adults	27,360 surgeries	CDC/NHSN SSI (incl. complex SSI)	NLP and ML	XGBoost	Administrative data Diagnosis codes Vital parameters Medication prescriptions Free‐text clinical notes	Sens: 83.8% Spec: 97.4% PPV: 74.3% NPV: 98.5% AUROC: 0.906 AUPRC: 0.637 F1 score: 0.788
SSI	Flores‐Balado [47]	2023	Semi‐automated	Development and validation + Clinical implementation	2014–2021	Spain	Surgical (THA), adults	7444 surgeries	ECDC SSI	NLP and ML	XGBoost	Administrative data Clinical chemistry Microbiology results Medication prescriptions; Free‐text clinical notes	Sens: 99.2% Spec: 91.0% PPV: 19.5% NPV: 99.9% Acc: 91.3% WR: 89.0% AUROC: 0.989 F1 score: 0.32
SSI	Cho [64]	2024	Semi‐automated	Development and validation	2013–2014	Korea	Surgical (colon), adults	1652 surgeries	CDC/NHSN SSI	ML	Rule‐based + NN	Administrative data Clinical chemistry Microbiology results Medication prescriptions Diagnosis codes Surgical procedures Radiology procedures Procedure codes NHSN SSI risk score	Sens: 93.9% Spec: 88.0% PPV: 28.9% NPV: 99.6% WR: 83.9%
UTI	Bouzbid [50]	2011	Fully automated	Development and validation	2000–2006	France	ICU, adults	1,499 patients	HELICS UTI	N/A	Rule‐based	Administrative data Microbiology results	Sens: 89.9% Spec: 96.5% PPV: 70.4% NPV: 99.0%
UTI	De Bus [51]	2014	Semi‐automated	Development and validation	2011–2012	Belgium	ICU, adults	876 admissions	CDC/NHSN UTI	N/A	Rule‐based	Administrative data Microbiology results Vital parameters Medication prescriptions	Sens: 80% Spec: 99.9% Acc: 77.8% Kappa: 0.80
UTI	Redder [52]	2015	Fully automated	Validation	2010–2014	Denmark	General	925 patients	CDC/NHSN UTI	N/A	Rule‐based	Administrative data Microbiology results Medication prescriptions	Sens: 78% Spec: 93% PPV: 88% NPV: 87%
UTI	Condell [65]	2016	Fully automated	Development and validation	2007	Denmark	General	1980 patients	CDC/NHSN UTI	N/A	Rule‐based	Administrative data Microbiology results	Sens: 50.0% Spec: 94.2%
UTI	van der Werff [39]	2021	Fully automated	Development and validation	2010–2012	Sweden	General	16,712 episodes from 15,986 admissions	ECDC UTI‐A CDC/NHSN UTI	NLP	Rule‐based	Administrative data Microbiology results Vital parameters Diagnostic codes Free‐text clinical notes	*ECDC UTI* Sens: 66.7% Spec: 99.7% PPV: 71.9% NPV: 97.7% AUROC: 0.832 *CDC UTI* Sens: 65.3% Spec: 99.6% PPV: 63.7% NPV: 99.6% AUROC: 0.825
CAUTI	Branch‐Elliman [36]	2015	Fully automated	Development and validation	2013	United States	General, veteran adults	2821 indwelling urinary catheter days	CDC/NHSN CAUTI	NLP	Rule‐based	Administrative data Microbiology results Vital parameters Free‐text clinical notes	Sens: 65.0% Spec: 99.6% PPV: 54.2% NPV: 99.7%
CAUTI	Van der Werff [39]	2021	Fully automated	Development and validation	2010–2012	Sweden	General	16,712 episodes from 15,986 admissions	ECDC CAUTI‐A CDC/NHSN CAUTI	NLP	Rule‐based	Administrative data Microbiology results Vital parameters Diagnostic codes Clinical notes	*ECDC CAUTI‐A* Sens: 59.5% Spec: 99.8% PPV: 62.9% NPV: 99.7% AUROC: 0.796 *CDC CAUTI* Sens: 54.2% Spec: 99.6% PPV: 44.8% NPV: 99.7% AUROC: 0.769
HAP	Bouzbid [50]	2011	Fully automated	Development and validation	2000–2006	France	ICU, adults	1499 patients	HELICS pneumonia	N/A	Rule‐based	Administrative data Medication prescriptions	Sens: 93.1% Spec: 63.5% PPV: 23.2% NPV: 98.7%
HAP	De Bus [51]	2014	Semi‐automated	Development and validation	2011–2012	Belgium	ICU, adults	876 admissions	CDC/NHSN Pneumonia	N/A	Rule‐based	Administrative data Microbiology results Vital parameters Radiology	Sens: 77% Spec: 99% Acc: 72.4% Kappa: 0.70
HAP	Wolfensberger [66]	2018	Fully automated	Validation	2016	Switzerland	General, adults	6064 patients	HELICS HAP	N/A	Rule‐based	Administrative data Diagnosis codes	Sens: 59% Spec: 98% PPV: 35% NPV: 99%
HAP	Wolfensberger [67]	2019	Semi‐automated	Development and validation	2017	Switzerland	General, adults	2091 patients	ECDC nvHAP	N/A	Rule‐based	Administrative data Vital parameters Clinical chemistry Procedures	Sens: 97.5% Spec: 100% PPV: 100% NPV: 99.2% Acc: 99.4%
VAP	Klompas [68]	2008	Semi‐automated	Development and validation	2006	United States	ICU, adults	459 patients	CDC/NHSN VAP	N/A	Rule‐based	Administrative data Microbiology results Vital parameters Clinical chemistry Radiology results Ventilator settings	Sens: 95% Spec: 100% PPV: 100% NPV: 99.8%
VAP	Kaiser [55]	2014	Semi‐automated	Development and validation	2009–2010	Netherlands	ICU, adults	553 patients	CDC/NHSN VAP	N/A	Rule‐based	Administrative data Microbiology results Vital parameters Clinical chemistry Medication prescriptions Radiology results Ventilator settings	Sens: 91.3% Spec: 100% PPV: 100% NPV: 99.8%
CDI	Dubberke [69]	2012	Fully automated	Development and validation	2005–2006	United States	General	1767 patients	CDC/NHSN CDI	N/A	Rule‐based	Administrative data Microbiology results	Sens: 92% Spec: 99% Kappa: 0.90
CDI	van der Werff [46]	2022	Fully automated	Development and validation	2011–2013	Sweden	General	180,715 episodes from 179,131 admissions	ECDC GI‐CDI	NLP	Rule‐based	Administrative data Microbiology results Clinical notes	Sens: 99.2% Spec: 100% PPV: 97.8% NPV: 100% AUROC: 0.996

Abbreviations: Acc, accuracy; AUPRC, area under the precision–recall curve; AUROC, area under the receiver operating characteristic curve; BSI, bloodstream infection; CAUTI, catheter‐associated urinary tract infection; CDC, Centers for Disease Control and Prevention; CDI, *Clostridioides difficile* infections; CLABSI, central line‐associated bloodstream infection; CRBSI, catheter‐related bloodstream infection; CRI, catheter‐related infection; CVC, central venous catheter; ECDC, European Centre for Disease Prevention and Control; HAP, hospital‐acquired pneumoniae; HELICS, Hospitals in Europe Link for Infection Control through Surveillance; ICU, intensive care unit; ML, machine learning; N/A, not available or not applicable; NHSN, National Healthcare Safety Network; NLP, natural language processing; NN, neural network; NNIS, National Nosocomial Infection Surveillance; NPV, negative predictive value; nvHAP, non‐ventilator‐associated hospital‐acquired pneumoniae; PPV, positive predictive value; Sens, sensitivity; Spec, specificity; SSI, surgical site infection; THA, total hip arthroplasty; TKA, total knee arthroplasty; UTI, urinary tract infection; VAP, ventilator‐associated pneumoniae; WR, workload reduction.

^a^ Size of validation cohort in case of multiple cohorts present and, if applicable, when extrapolated results are presented the size of this cohort.

^b^ In case of multiple algorithms present, for best performing and/or most relevant algorithm.

### Bloodstream infections and catheter‐related bloodstream infections

BSI have often been a target for automated surveillance using rule‐based algorithms. Based on administrative data, microbiology results and sometimes complemented with the use of vital parameters and medication prescriptions, good performance has been obtained, with a sensitivity of 80%–100% and specificity of 94%–100%, compared to manual surveillance [49, 50, 51, 52]. In relation to this satisfactory performance, probably no effort has been made to use AI‐based methods. No other blood or antigen tests were used as potential sources of data in these studies, and the use of such types of data could be further explored.

In addition, CRBSI, BSI related to the use of intravascular catheters, are common targets for automated surveillance, both fully and semi‐automated. A recent review showed that rule‐based algorithms using structured data, such as administrative data (healthcare admission and discharge data), microbiology results and symptom data, have already performed well, with a pooled sensitivity of 89% and specificity of 83% [70]. These algorithms mostly target the Centers for Disease Control and Prevention (CDC) central line‐associated BSI definition. One study targeting the European Centre for Disease Prevention and Control CRI3‐central venous catheter definition using similar data sources also demonstrated good performance, with a sensitivity of 88% and a specificity of 100% [57]. However, studies using AI‐based methods have not yet been conducted. Nevertheless, this HAI could be a good target for AI‐based methods to improve algorithm performance, for example, by using NLP and/or ML to improve the detection of intravascular catheters, symptoms in free‐text notes and identifying other sites of infection.

### Sepsis

Nosocomial sepsis is an overlooked target for surveillance and is poorly defined, as HAI criteria largely focus on specific sites of infection or microbiologically confirmed infections. The CDC developed the Adult Sepsis Event definition for automated surveillance using EHR data [43, 58]; however, it is not specifically designed to capture nosocomial sepsis. The Adult Sepsis Event definition has 70% sensitivity, 98% specificity and 70% positive predictive value for the accurate classification of sepsis [58]. A limitation of the CDC definition is that it differs from the Sepsis‐3 clinical criteria [71], which is the current international definition of sepsis, by not assessing organ dysfunction according to the Sequential Organ Failure Assessment score [72]. The CDC definition is dependent on treatment interventions for two organ dysfunction criteria (cardiovascular and respiratory), biasing sepsis surveillance towards patients eligible for aggressive treatment and those with access to intensive care unit (ICU) care, thereby limiting generalizability to all hospitalized patients. In a recent study from Sweden, an automated rule‐based algorithm to detect sepsis according to the Sepsis‐3 definition using EHR data showed a sensitivity of 89%, a specificity of 99% and a positive predictive value of 88% [59]. Restricting the analyses to hospital‐onset sepsis resulted in a sensitivity of 79%, a specificity of 99.7% and a positive predictive value of 88%. Both studies demonstrated that administrative data, microbiology results, vital parameters, clinical chemistry, antibiotic prescriptions and diagnostic codes are important sources of data for sepsis algorithms [58, 59].

### Surgical site infections

SSI is one of the most studied targets of automated surveillance. The large volumes of procedures and the relatively low incidence of SSI for some surgery types provide potentially high gains for automated surveillance. Traditionally, most automation efforts have relied on rule‐based semi‐automated surveillance using structured data such as administrative data, surgical procedures, microbiology test results and antibiotic prescriptions to identify patients with a possible SSI [23, 60, 61, 62]. AI‐based methods have been studied as alternatives or complements to this approach. For example, Wu et al. [63] developed ML‐based algorithms on structured administrative data complemented with free‐text notes to detect SSI after hip replacement, achieving over 90% sensitivity. In their study, the combination of administrative data and processed free‐text notes outperformed models based on only one form of data [63]. Flores‐Balado et al. also presented an NLP‐ and ML‐based approach for the detection of SSI after hip replacement in the setting of semi‐automated surveillance that achieved near‐perfect sensitivity and a workload reduction of approximately 90% [47], a figure comparable with rule‐based semi‐automated surveillance [61]. The added value of the NLP of free‐text data in addition to structured data will likely depend on the SSI target and the type of structured data employed. For example, Verberk et al. studied the added value of NLP in addition to a rule‐based algorithm relying on structured clinical data for the detection of SSI after colorectal surgery and found no improvement in terms of sensitivity or positive predictive value [40].

### Urinary tract infections and catheter‐associated urinary tract infections

UTI, including CAUTI, is another type of HAI for which automated surveillance using AI‐based methods is feasible. Based on administrative data, microbiology results, often use of medication prescriptions and sometimes vital parameters, good model performance has already been achieved for UTI. AI‐based methods are not often used for this purpose, although NLP has been utilized to detect CAUTI. Branch‐Elliman et al. described an algorithm for CAUTI detection using NLP that replaced the conventional chart review at a large urban hospital. Although the identification of CAUTI by the algorithm was successful, reliance on the quality of local documentation practices was seen as a potential obstacle to the generalizability and real‐life application of the algorithm [36]. A solution could be standardization of medical chart documentation practices, but this might be difficult to achieve. Another study employing NLP‐based algorithms to detect CAUTI and UTI in general validated and compared five fully automated algorithms for detecting healthcare‐associated UTI using different data sources [39]. Here the inclusion of symptoms from the free text, compared to microbiological data alone, improved the positive predictive value from 38.9% to 71.9%.

### Hospital‐acquired pneumonia and ventilator‐associated pneumonia

Although studies have been published exploring AI, including DL techniques, for the early detection of HAP [73] and VAP [74], applications of these techniques for surveillance purposes have not yet been described. For VAP, already good performing semi‐automated algorithms have been developed using administrative data, microbiology results, vital parameters, clinical chemistry, radiology results and ventilator settings with or without the use of antibiotic prescriptions. However, AI‐based methods can also be explored to determine whether fully automated surveillance is possible. Recently, a review by Wolfensberger et al. on the automated surveillance of HAP and non‐ventilator‐associated HAP (nvHAP) was published [75]. In that publication, the results of the performance and validation of automated surveillance were reviewed for 13 studies, 12 of which presented algorithms for fully automated surveillance and one for semi‐automated surveillance. The algorithms applied to limited structured source data, such as diagnostic codes at discharge, were outperformed by algorithms which included clinical data (solely or in combination with diagnostic codes) like data on microbiology, antibiotics, radiology, fever, leukocyte counts and deteriorating oxygen levels. NLP has been used in some studies to analyse unstructured clinical data. Overall, the performance of the algorithms was mostly moderate, with sensitivities ranging between 40% and 99% and specificities ranging from 58% to 98%. However, a sound methodology to evaluate performance is lacking. Due to the heterogeneity in definitions, reference standards and limitations in the study population, the results remain inconclusive. However, AI was noted as a technique that could potentially contribute to improving algorithm performance for automated nvHAP surveillance [75]. The improvement of algorithms with additional data sources, including symptoms (such as coughing) or auscultation, could be exploited using AI.

### Clostridioides difficile *infections*

Although CDI is one of the most easily automated HAIs, few studies on automation efforts compared to manual surveillance have been published. However, fully automated surveillance has probably been implemented more widely than for any other HAI, as this largely depends on access to microbiological culture information, which is often available. Existing studies confirm that CDI is an easy target for fully automated surveillance and show excellent performance, achieving sensitivity and specificity between 92% and 100% [46, 69]. NLP was used in one study, but it did not provide much added benefit in a setting where testing for CDI is primarily performed when there are symptoms. The algorithm using only microbiological data for CDI surveillance also performed well [46].

## Moving beyond surveillance: Early identification and prediction of HAI and severe infections

In addition to employing EHR data for epidemiological HAI surveillance, increasing attention is being paid to early warning systems (EWS) or prediction of HAI, with the aim of initiating prompt treatment or performing targeted prevention for precision medicine. Consequently, there is increased interest in developing automated early prediction models for HAI using ML. If models with sufficient accuracy and timeliness in predicting events can be obtained, this will help move IPC programmes towards personalized IPC, facilitating individualized interventions to reduce the risk of future HAI. The use of clinical data sources for real‐time EWS or risk prediction poses additional challenges to both the quality and timeliness of data sources, as well as the complexity of the methodology and its ability to incorporate time‐varying predictors. This is still a young research field in which methods are in continuous development.

ML and NLP can be employed in the same processes as automated surveillance, such as extracting structured information from unstructured information or classifying information. However, ML and NLP are especially used to harness the vast amount of information available in EHR data before HAI onset and can utilize this information to predict *future* HAI risk. Studies have focused on identifying whether accurate prediction (of risk) at static time points is possible with mixed success but have often not explored how long before (early) HAI onset prediction is possible [44]. Although there was heterogeneity among the studies in terms of the type of data used, settings, model designs and assessed measures, the models showed moderate‐to‐good performance. ML models using EHR data from the first 24–48 h after hospital admission were developed to predict future healthcare‐associated UTI, which showed performance of AUROC (area under the receiver operating characteristic curve) of 0.71–0.77 [76]. Recently, a meta‐analysis pooled data from 10 static prediction models using supervised learning for VAP [77]. The pooled AUROC, sensitivity and specificity for VAP were 0.88, 0.72 and 0.90, respectively. Most models have a low positive predictive value, indicating that they are likely to generate many false warning signals when implemented in clinical practice. This is a concern, as it might result in alert fatigue. There is also a trade‐off among performance, explainability and transparency, where DL models may have higher performance than other ML or non‐ML models at the cost of explainability in clinical settings [76]. To our knowledge, no model for predicting HAI has been implemented or evaluated in clinical practice.

Early prediction models have been published for patients at risk of sepsis [3, 78–82]. In a 2020 review, 28 papers reporting the performance of 130 models were included [78]. Most models were developed for the ICU, and only three have been implemented with varied results. The diagnostic accuracy varied from 0.61 to 0.99, and varying sepsis definitions precluded pooling of results. Despite the heterogeneity in settings, patient populations, sepsis definitions and model designs, the models showed moderate to very good performance. In some studies, the prediction time before the onset of infection was determined, which demonstrates that early prediction was possible, although there was a large variation in the range of 0–40 h before the onset of sepsis. Additionally, it is often difficult to determine the time of actual onset of sepsis [82]. Only a few prediction models have been implemented in clinical practice and have shown varied effects on patient outcomes. A prospective, multisite cohort study examined the association between patient outcomes and provider interaction using a deployed sepsis alert system called the targeted real‐time EWS (TREWS) [79]. TREWS consists of a mixture model of Cox proportional hazards models to account for patient heterogeneity. During the study, 590,736 patients were monitored using TREWS across five hospitals. A total of 6,877 patients with sepsis were identified by the alert before the initiation of antibiotic therapy, and when the alert was confirmed by a provider within 3 h of the alert, in‐hospital mortality, organ failure and length of stay were reduced compared with patients whose alerts were not confirmed by a provider. In contrast, a study that assessed the performance of the EPIC sepsis model (EPIC is a provider of an EHR system) showed a sensitivity of only 33% and a positive predictive value of 12% in identifying patients with sepsis [80]. A study assessing the EPIC sepsis model in nine different US hospitals showed an AUROC performance varying from 0.55 to 0.73 depending on the hospital [81]. Notably, performance can be influenced by heterogeneity in patient populations as well as differences in the EHR data structure, highlighting the importance of external validation [83]. The evaluation of real‐world performance should preferably be performed using the most robust study design, such as a randomized controlled trial (RCT). In contrast, to achieve the highest accuracy and continuous development and adaptation of AI screening models, an RCT may not be the most effective way to assess performance. One RCT with a small sample size showed that the length of stay and in‐hospital mortality significantly decreased with the use of an ML model compared to existing sepsis detectors [84]. As most studies have not distinguished between community‐ and hospital‐onset sepsis, it is difficult to assess the impact of these systems on patients at risk of developing nosocomial sepsis.

## Fundamentals of HAI surveillance—what requirements should be met for successful (AI‐supported) automated surveillance

Developments in AI are rapid, and more are expected in the coming years. However, irrespective of the method of surveillance automation, some basic principles need to be ensured to retrieve reliable and valid HAI surveillance and prediction results by (AI‐supported) automated systems. In the automated surveillance of HAI, precise definitions, standardized protocols and consistent data quality are pivotal [85]. Automated systems rely on clear definitions to identify cases accurately, standardized protocols to ensure interoperability and consistent data quality to enhance reliability. These elements form the backbone of automated HAI surveillance, which enables reproducible and timely detection. Table 3 lists the important requirements and implications for technical capabilities, governance and practical considerations that should be carefully considered for the successful development and implementation of (AI‐supported) automated surveillance in individual healthcare centres or surveillance networks.

**Table 3:** *Requirements for successful (AI‐supported) automated surveillance or prediction*.

Requirement	Explanation	Implications and targets
Accuracy	The capability of AI models to accurately identify and classify HAI with high precision	– Accurate performance metrics (sensitivity, specificity, etc.) – Validation against gold standard or expert judgements for accuracy – Use of separate representative test datasets for robust development – Cross‐validation techniques to assess generalization
Comparability of algorithm performance	Comparability of algorithm performance between hospitals participating in a surveillance network	– Standardized evaluation protocols and datasets for consistent over time, between centres comparison – Benchmarked datasets representing real‐world HAI scenarios
Timeliness	The capability of AI models to provide prompt and timely results, insights and alerts for efficient HAI detection	– Timely and continuous data processing for prompt detection and response – Implementing effective alerting mechanisms for timely notifications – Enabling rapid response to positive HAI cases for better patient management
Reproducibility	The ability to replicate and verify research findings and methodologies in AI model development and evaluation	– Providing open access to code and FAIR data for transparency and replication – Transparent reporting of experimental setups and performance metrics – Conducting prospective studies in real‐world clinical environments
Accessibility of data and analysis techniques	Ensuring technology and data are easily available, usable and understandable to ensure availability of AI and automation tools in various healthcare settings or at the level of the coordinating centre in surveillance networks	– Transparent and interpretable models for better understanding – User‐friendly interfaces for healthcare professionals – Interoperable data formats for seamless analysis – Training and support programmes for effective usage
Data privacy and security	Ensuring the confidentiality and protection of patient data used in AI model development and evaluation	– Compliance with data privacy regulations for patient confidentiality – Encryption and secure storage to safeguard sensitive information – Role‐based access control to limit data access to authorized personnel only – Regular data audits and risk assessments for identifying vulnerabilities to data security breaches – Employ suitable methods of data minimalization, pseudonymization and anonymization where necessary
Trustworthiness and acceptance by clinicians and other stakeholders at hospital or public health level	The level of support and approval from healthcare professionals in integrating AI into clinical practice for HAI detection	– Involvement of healthcare professionals and patients and AI, ethical and legal experts in the developmental, validation and maintenance phase for reviewing data and outcomes, utilization strategy, addressing needs and concerns – Transparent communication of AI results, technology's potential impact on healthcare benefits and uncertainties or limitations for building trust – Demonstrated efficacy in improving patient outcomes and IPC measures – Ongoing training and support to address concerns and ensure effective usage – Compliance with ethical and privacy considerations and AI regulatory requirements – Cost‐effective implementation in different healthcare facilities
Transparency	The practice of openly sharing research findings, methodologies, decision‐making and code to foster collaboration and transparency	– Sharing datasets and code in public repositories for validation and replication – Providing detailed documentation of methodologies and parameters for better understanding – Present research results in open access journals and meetings – Creating educational resources to promote broader knowledge sharing and understanding the basics of AI specific to HAI surveillance and innerworkings of algorithms to increase intelligibility of model decisions and outcomes for professionals and patients
Governance and accountability	Clear definition of roles and responsibilities for developers/third parties, hospitals and public health	– Legal guidance on liability – Introduction of standards or benchmarks to decide on applicability, data protection, transparency and bias minimization, safety and quality risks (e.g., guidelines for high‐quality diagnostic and prognostic applications of AI in healthcare) – Standard reporting framework comparable to TRIPOD + AI (transparent reporting of a multivariable prediction model for individual prognosis or diagnosis) – Local quality management and monitoring plan for initial and annual appraisal of algorithm and software – Local education for competence in use and appraisal – Transparency among all stakeholders in performance and issues

Abbreviations: AI, artificial intelligence; FAIR, Findability, Accessibility, Interoperability and Reusability; HAI, healthcare‐associated infections; IPC, infection prevention and control.

*Source*: Based on van Mourik et al. [2], van Mourik et al. [20], Collins et al. [86], Insight—ALTAI [87], van Rooden et al. [88], Reddy et al. [89].

Practical implementation of such systems is difficult and requires considerable investment. Harmonizing the requirements for the automation of surveillance and AI used in surveillance presents notable challenges. Many of these have been described for other purposes such as antimicrobial resistance (AMR) [90]. Similar to AMR, the first question is whether AI is the correct solution to the problem. Furthermore, continuous adaptation and flexibility in incorporating emerging IT solutions are necessary to ensure the effectiveness and sustainability of AI‐supported surveillance initiatives. By remaining attuned to evolving IT capabilities and embracing a proactive approach to addressing emerging challenges, healthcare systems can leverage the full potential of AI to enhance HAI surveillance and improve patient outcomes in the dynamic landscape of IPC. In this context, it is crucial for automated surveillance algorithms to successively integrate information from other sources. For example, the link between genomic data and automated systems for the detection of HAI and clusters of HAI has not been widely established. By leveraging genomic sequencing technologies, automated systems can analyse microbial genomes, enabling the precise identification of pathogens and reconstruction of transmission pathways. This approach enhances the accuracy and timeliness of HAI detection, facilitating targeted interventions to prevent outbreaks or, more broadly, the incidence of HAI [91]. Additionally, because data from EHR systems are often unstructured with variable formats and registration methods, creating interoperable data in a machine‐readable format is important for AI applicability [92]. Adherence to the FAIR (Findability, Accessibility, Interoperability and Reusability) principles is particularly important for the re‐use of these data [93]. Synergy can be achieved by making routine care data more widely available in conjunction with FAIR principles, which increases the amount of source data available for analyses to improve model development. Efficiency in meeting prerequisites, such as increased digital literacy and essential knowledge development for the secondary use of source data in specific domains, can be enhanced when applied to multiple related purposes like HAI and AMR.

Irrespective of the methodology, trust, use and quality of the data are vital for application, correct results and, hence, improvement in the quality of care. Systematic reviews on automated nvHAP surveillance [75] and on AI and ML for automated surveillance of a broader range of HAI [44] exemplify the lack of sound methodology, including validation, even in studies with good reference samples and accepted case definitions. Consequently, insufficient insight can be gained into the quality of the methodology and surveillance results. This impedes revealing the generalizability and potential of NLP or AI and, therefore, trust building and, potentially, the achievement of surveillance aims. Checklists such as ‘TRIPOD + AI’ could help close this gap [86].

As shown in Table 2, nearly all studies reviewed herein were performed in research settings. When moving towards implementation, regulatory requirements should be carefully considered in the context of re‐using or combining data and for when or in which manner AI can be applied. Several frameworks and checklists have been developed that could be supportive when developing (AI‐supported) surveillance systems [87, 94].

## Monitoring quality of automated surveillance or prediction systems

Assessing and monitoring the quality of automated surveillance or prediction systems is essential to ensure their reliability over time and as clinical practices or underlying data sources change. Quality assurance is important during the development and implementation as well as after the implementation of the system and demands attention to various factors.

During the developmental stage, one of the important tasks is to define the markers of performance, that is, what is minimally expected from the system or algorithm (e.g., high sensitivity, trade‐off between sensitivity and specificity). The choice of performance markers depends on the intended use of the system and the extent to which human verification will validate the outcomes. Second, because algorithms are dependent on available source data for training, testing and validation, these data should be of high quality (accurate, complete and consistent), unbiased (collected from diverse and representative samples) and representative of the intended application (reflecting the real‐world population and conditions) in order to estimate the expected performance according to the predefined performance markers [89, 95]. For HAI surveillance, the data need to be representative for either a single hospital or hospitals within a surveillance network, equivalent to the intended application of the algorithms, and represent the current patient populations, routine care procedures and routine care diagnostics, and data availability. Potential caveats that may affect algorithm performance have been described for laboratory data [90, 96], underscoring the importance of digital literacy and domain knowledge when re‐using data.

In addition, a systematic assessment of the suitability of these methods for local clinical practice is essential. In their validation study, Verberk et al. demonstrated that differences in microbiological culture practices affect the performance of algorithms for detecting SSI; pre‐emptively assessing such differences when validating or implementing methods will improve model performance [97]. Understanding the information captured in the registered data, meaning of data (including missing data) and interpretation of (combined) data elements requires knowledge of clinical reasoning and registration procedures [96].

Furthermore, a comparison of results, whether between hospitals or within a single hospital, is important. However, comparability across hospitals is not necessarily ensured by applying the same algorithm, as this depends on the heterogeneity of routine care data. Recently, it was shown that differences in patient population, ward attribution and treatment regimens between hospitals had an impact on the development of predictive models for sepsis and acute kidney injury [98]. As a result, the generalized algorithms had worse outcomes than the locally developed algorithms. This is a clear example illustrating that with AI, automated surveillance might require local validation and competence to understand (centrally) developed models in the context of the underlying source data, their applicability and the identification of unexpected results. To prevent local validation procedures from becoming too costly and resource‐intensive, it is recommended to develop a framework for efficient validation [99].

After implementation, to ensure comparability over time, regular appraisal is important to detect influences that have a negative impact on model performance, such as performance drift. Performance drift is defined as the underperformance of an algorithm or model due to disparities between the source data for algorithm development and the data on which the algorithm is applied. Finlayson et al. presented a list of the underlying causes of performance drift that can be classified into changes in medical and technology practices, changes in population or (healthcare) settings and changes in the behaviour of medical specialists or patients [100]. There are different methods for detecting performance drift, including continuous monitoring of the predictive performance or monitoring the confidence scores of an ML model's predictions, the latter of which indicates how sure the ML model is that output was correct. Consequently, for reliable surveillance and prediction results that are comparable over time, continuous feedback on the performance of the model is essential, as well as constant retraining and updating of the ML models that were deployed. Again, clinical expertise is indispensable; vigilant clinicians need to report changes in clinical practice and concerns about algorithm performance, in addition to technical oversight, to avoid performance drift [100, 101]. This highlights the importance of domain, medical and technical knowledge of the EHR structure when re‐using routine care data in algorithm applications [102].

In addition to the ongoing monitoring of the performance of the chosen system, the maintenance of models and data infrastructure will be an ongoing process to accommodate changes in the underlying data structure, data models and protocol changes in the case of HAI surveillance. In an ideal situation, a quality management system is used to systematically and pre‐emptively monitor data quality and meaning, and procedures should be in place to verify whether clinical practice or document habits change over time [2].

## Conclusions and future perspective

The field of AI and automated surveillance (i.e., detection) or (early) prediction of HAI and other infections is evolving. The key takeaway messages are as follows:

- AI‐based methods have been applied less frequently in automated surveillance and more frequently for (early) prediction, particularly for sepsis. Perhaps this is, in part, the result of the increased complexity of early prediction as opposed to the retrospective classification performed in surveillance.
- AI‐based methods have the potential to improve the prediction (screening) of patients at risk for HAI, contributing to personalized medicine.
- AI‐based methods should be explored more to support and improve the various steps in the automated surveillance process.
- NLP, with or without LLM's, can be particularly useful for extracting information from unstructured free‐text notes in EHR data.
- Publications on the implementation and evaluation of (AI‐supported) automated HAI surveillance and prediction of HAI in clinical practice remain scarce.
- The successful development and implementation of (AI‐supported) HAI surveillance and prediction systems requires considerable investment to address technical capabilities, governance, practical and regulatory considerations, and quality monitoring.
- Irrespective of the methodology, basic requirements, including data quality assurance, unbiased and representative data, and adherence to standardized protocols, are crucial for reliable (AI‐supported) HAI surveillance and prediction results.
- The evaluation of accuracy and performance in multiple settings is crucial for achieving generalizable results.
- The continuous monitoring and updating of models, including AI‐supported models, are essential for maintaining performance and adapting to changes in clinical practices and data sources.

By exploiting the possibilities of AI and data science, HAI surveillance and prediction can offer more objective and timely rates and predictions for the benefit of patients, healthcare personnel, healthcare institutes and society. The successful application of AI in automated HAI surveillance and prediction can be transferred to other purposes, such as AMR surveillance. The adaptation and expansion of these models can transform infectious disease monitoring and prediction into diverse healthcare domains. Advances in data science and AI in other medical fields may, in turn, strengthen applications in infectious diseases, both in terms of methodology and development of a sustainable data infrastructure.

Efforts should be directed towards ensuring the adaptability of AI models across different languages and settings. Multilingual capabilities would enhance the global applicability of these systems, promoting broader impact and utilization. Current developments in generative AI, particularly LLMs, hold promise in contributing to this, apart from their potential to generate valuable textual content. Integrating these capabilities into surveillance and prediction systems can enhance the reporting, documentation and communication in healthcare settings.

Currently, HAI surveillance relies on standardized definitions, which can be limited by the availability and quality of patient data and documentation. As discussed, NLP can extract relevant information from unstructured clinical notes, thereby limiting the need to compromise on existing definitions. However, LLMs and generative AI can support and improve the documentation and quality of data beforehand. By integrating and analysing improved data from multiple sources, ML models can potentially identify new patterns and correlations that improve the accuracy of HAI detection, especially for complex HAI such as CRBSI and nvHAP. Furthermore, AI systems can continuously learn and adapt to new data, which could potentially refine HAI definitions over time and even lead to the development of personalized criteria based on individual patient characteristics. These advancements may lead to more precise and effective HAI surveillance, ultimately improving patient outcomes. Exploring new modalities and techniques, such as monitoring patient movement, may present innovative opportunities for surveillance and prediction. The integration of predictive information from external sources, such as wearable sensors and computer vision monitoring, could also provide additional possibilities to AI/ML models. Despite the potential offered by these new modalities, access to high‐quality labelled data for training AI/ML models remains a challenge. The success of these models relies heavily on the richness and diversity of the datasets. Collaborative efforts across stakeholders to build comprehensive datasets are pivotal for refining surveillance and prediction models and ensuring their accuracy. Multidisciplinary synergy can also lead to a more efficient use of resources and relevant knowledge development.

Synergy can also be achieved by linking HAI and AMR surveillance. As they are closely related with an overlap in source data relevant for automation, effective HAI surveillance systems should strive to incorporate AMR data to provide a comprehensive understanding of the infection burden and resistance patterns. Automated surveillance systems and AI can improve the detection of AMR, predict susceptibility profiles and detect outbreaks, thereby supporting IPC measures and timely and appropriate antimicrobial therapy. This integration can improve patient outcomes and aid the fight against AMR, a growing global health threat.

It is important to address the opacity of existing black‐box AI models. Embracing explainable AI models enhances interpretability, promotes trust and aids healthcare professionals in understanding model outcomes. However, recognizing concerns regarding sensitive information embedded in the model parameters is also crucial. Future developments should prioritize robust privacy‐preservation techniques to mitigate potential risks. Achieving a balance between the benefits and privacy concerns of AI‐supported solutions is vital. Robust privacy policies, transparency and secure data‐handling mechanisms should be integral components of system design.

The implementation of AI‐based methods in clinical practice using EHR data is a relatively new field that requires considerable effort, like data integration, algorithm development and validation and training of users. Therefore, it is important to evaluate the impact of implemented systems. RCTs are not always feasible; therefore, exploring alternative study designs and methodologies is essential for robust assessments and evidence generation. In addition, active engagement of end users, including healthcare professionals, in the development and deployment phases of surveillance and prediction systems is essential. These medical insights will contribute to system refinement, usability and overall effectiveness.

## Author contributions

**Suzanne D. van der Werff**, **Stephanie M. van Rooden**, **Aron Henriksson**, **Michael Behnke**, **Seven J. S. Aghdassi**, **Maaike S. M. van Mourik** and **Pontus Nauclér**: Conceptualization; writing—original draft; writing—review and editing. **Suzanne D. van der Werff**, **Aron Henriksson** and **Maaike S. M. van Mourik**: Visualization. **Suzanne D. van der Werff**: Project administration. **Pontus Nauclér**: Supervision.

## Conflict of interest statement

PN and SvdW are involved in a company that works on automated surveillance for adverse events. The other authors have no conflicts of interest to declare.

## Funding information

DFG, Charité Universitätsmedizin—Berlin and Berlin Institute of Health at Charité (BIH). RZN ABR network Utrecht through a grant from the Dutch Ministry of Healthcare, Welfare and Sport (Grant number 331724). Region Stockholm (ALF Grant 2019‐1054), the Swedish Research Council (VR Grant 2021‐02271) and Sweden´s Innovation Agency (Vinnova Grant 2021‐02699)

## Data Availability

Data sharing is not applicable to this article as no datasets were generated or analysed during the current study.
